# Aggregated SOD1 causes selective death of cultured human motor neurons

**DOI:** 10.1038/s41598-018-34759-z

**Published:** 2018-11-06

**Authors:** Chen Benkler, Alison L. O’Neil, Susannah Slepian, Fang Qian, Paul H. Weinreb, Lee L. Rubin

**Affiliations:** 1 Department of Stem Cell and Regenerative Biology, Harvard Stem Cell Institute, Harvard University, Cambridge, MA USA; 2 Biotherapeutics and Medicinal Sciences Department, Biogen Inc., Cambridge, MA USA

## Abstract

Most human neurodegenerative diseases share a phenotype of neuronal protein aggregation. In Amyotrophic Lateral Sclerosis (ALS), the abundant protein superoxide dismutase (SOD1) or the TAR-DNA binding protein TDP-43 can aggregate in motor neurons. Recently, numerous studies have highlighted the ability of aggregates to spread from neuron to neuron in a prion-like fashion. These studies have typically focused on the use of neuron-like cell lines or neurons that are not normally affected by the specific aggregated protein being studied. Here, we have investigated the uptake of pre-formed SOD1 aggregates by cultures containing pluripotent stem cell-derived human motor neurons. We found that all cells take up aggregates by a process resembling fluid-phase endocytosis, just as found in earlier studies. However, motor neurons, despite taking up smaller amounts of SOD1, were much more vulnerable to the accumulating aggregates. Thus, the propagation of disease pathology depends less on selective uptake than on selective response to intracellular aggregates. We further demonstrate that anti-SOD1 antibodies, being considered as ALS therapeutics, can act by blocking the uptake of SOD1, but also by blocking the toxic effects of intracellular SOD1. This work demonstrates the importance of using disease relevant cells even in studying phenomena such as aggregate propagation.

## Introduction

ALS is a progressive neurodegenerative disease in which the loss of motor neurons (MNs) leads to paralysis and ultimately death due to respiratory failure- usually within 2–5 years of symptom onset. Typically starting late in life, ALS progresses along neuroanatomical pathways meaning symptoms often begin in one extremity and spread to the one closest to it, and so on, progressing through the central nervous system (CNS).

Despite extensive research, the underlying causes of ALS and the paths of neurodegeneration remain elusive. Some of the leading hypotheses include: glutamate-excitotoxicity, glutamate dependent and independent oxidative-stress, deficits in neurotrophic factors, mitochondrial dysfunction and neuroinflammation^1–4^. Another relatively new theory, that is rapidly gaining traction, is cellular toxicity caused by intracellular protein misfolding and aggregation^2,5–7^. Protein aggregation is a hallmark of many other neurodegenerative diseases as well. For example, in Alzheimer’s disease (AD), amyloid-beta and tau cause the hallmark plaques and tangles in the brains of patients, while in Parkinson’s disease (PD), alpha-synuclein aggregates are often found in the affected dopaminergic neurons^8–11^. In Huntington’s disease, the extended poly-Q repeats in the huntingtin protein make it very prone to aggregation, again resulting in the hallmark pathological feature of intracellular aggregates in striatal neurons^12–16^. Furthermore, for each disease, there appears to be pathological spread along anatomical pathways.

Because of this commonality among neurodegenerative diseases, it is not surprising that there has been increased interest in the potential prion-like behavior of aggregating proteins in ALS. However, unlike AD and PD, little is known about the potential involvement of protein aggregation in ALS pathophysiology and spread. Mutations in several genes (*SOD1*, *TARDP*, *FUS* and *OPTN*) are known to lead to protein aggregation and the development of ALS^17–20^. Interestingly, both the genetically-driven familial and the sporadic forms of ALS are clinically and pathologically similar, suggesting a possible common pathogenesis and a shared pathway of neurodegeneration. Indeed, SOD1 and TDP-43 protein aggregates, often associated with ubiquitin, can be found in postmortem spinal cord samples from both sporadic and familial ALS patients, as well as in animal models^21–23^.

Mutations in the gene coding for the protein superoxide dismutase 1 (SOD1) were among the first identified as being involved in ALS. It is not the loss of function of the enzyme that leads to the pathological outcome, but rather a gain of toxic function, with many of these mutations leading to the enzyme’s aggregation^5–7^. The SOD1 protein is ubiquitously expressed but, in ALS, it causes specific MN degeneration despite the presence of SOD1 aggregates not only in MNs, but also in the nuclei of ventral horn astrocytes, microglia, and oligodendrocytes of familial ALS, as well as sporadic ALS, patients^24^. There is experimental evidence supporting uptake and propagation of pathological conformations of both SOD1^21,25–28^ and TDP-43^29,30^, but the precise mechanism of SOD1 aggregate transfer is unknown. Consistent with the prion hypothesis, these proteins have been shown to induce a pathologic conformation on their natively folded counterparts in a template-directed manner^19,26,28^. Studies performed in ALS mice found that the injection of mutant SOD1 spinal cord homogenates into the spinal cords of G85R-YFP-SOD1 mice induces ALS like pathology that spreads through neuroanatomical pathways^25^. In another study, mutant and wild-type (WT) SOD1 proteins can transfer between MNs in a process that might be mediated through other cell types, possibly oligodendrocytes, which in turn, might even enhance the aggregate toxicity^21^. Recent work further suggests that exosomes and tunneling nanotubes may be responsible for cell-to-cell transmission^29,30^.

The reason why MNs specifically degenerate, while other cell types in the same niche remain healthy, is still not understood. Previous studies of SOD1-aggregates were restricted to cells that are biochemically very different from MNs such as the neuroblastoma cell line Neuro2A (N2A), the spinal cord-neuroblastoma hybrid cell line NSC-34, the adrenal gland tumor line PC12 and a Chinese hamster ovary (CHO) cell line^27,28,31,32^. Use of readily available cell lines instead of the exact cell type associated with the disease is common in neurodegeneration research.

To improve upon these models, we employed MNs produced from human pluripotent stem cells (hPSCs). Using these cells, we were able to extend the scope of the previous studies and to identify mechanisms pointing to MN cell type specificity. Here we show that SOD1 aggregates are specifically toxic to MNs although all cells in differentiated cultures take them up. Furthermore, we found that dividing cells such as the N2A and NSC-34 cell lines take up SOD1 aggregates much more readily then the MNs do, but are significantly less sensitive to aggregated SOD1-induced toxicity. These findings highlight the importance of characterizing disease phenotypes in the correct cell type to truly recapitulate the disease mechanism *in vitro*. Additionally, we investigated the use of anti-SOD1 antibodies, similar to those being considered for clinical use, to reduce the spread of SOD1 aggregates and found that anti-SOD1 antibodies decrease MN toxicity both by blocking uptake, as might be expected, but also by reducing the toxicity of already internalized aggregates.

## Results

### Cellular uptake of SOD1 Aggregates

**Figure 1:**
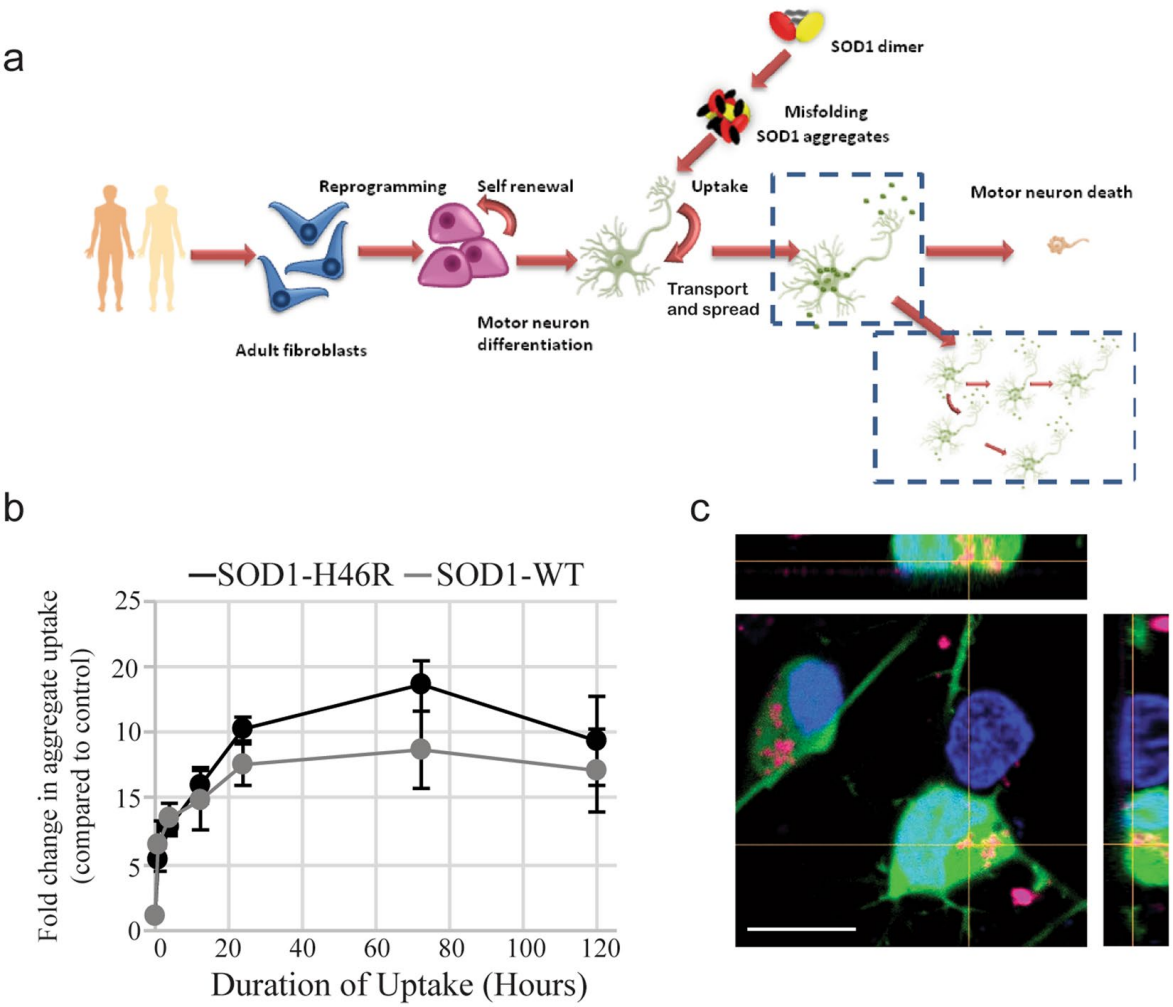
Mutant SOD1 aggregates are taken up by human hPSC-derived MNs. (**a**) Schematic representation of the experimental paradigm. (**b**) Bulk uptake of DyLight 650 labeled WT and SOD1^H46R^ aggregates measured by the 650 nm intensity in all cells in the culture by flow cytometry. Cells were first trypsinized to remove aggregates adhering to the cell surface. Fold change is calculated as the average intensity ± SD normalized to untreated control. A repeated measures one-way ANOVA showed the uptake curves are not significantly different (p = 0.124). (n = 3) **(c**) Representative confocal micrographs of DyLight 650 labeled SOD1^H46R^ aggregates (magenta) internalized by MNs (Islet1-GFP); Hoechst labeled nuclei are blue. Scale bar = 10 µm.

In patients, ALS causes selective MN degeneration, while other cell types in their immediate microenvironment are better preserved. Therefore, to evaluate the potential involvement of prion-like SOD1 aggregates in the disease, we found it particularly important to simulate the ALS niche as closely as possible. To this end, we differentiated hPSCs into MNs using a 3D spinner culture method that we have previously described (Fig. 1a)^33^. This method of differentiation reliably yields mature human MNs that, following dissociation and replating, are physiologically active and remain healthy without the need for a glial feeder layer. This differentiation method produces cultures that contain approximately 35% Islet1 positive motor neurons, as well as other types of neurons and some residual Ki67+ proliferating cells (data not shown).

We first validated our system by confirming that SOD1 aggregates are taken up by 2 neuronal cell lines, N2A and NSC-34, as previously reported. For this study, we chose to make SOD1 aggregates from one of the most common mutant forms (H46R), as well as from non-mutated (WT) SOD1. To create the aggregates, purified, endotoxin free recombinant SOD1 proteins were labeled with DyLight 650 and then aggregated as previously described^28^. As expected, both of the cell lines were able to take up the fluorescently labeled aggregated SOD1 (Supplementary Fig. S1a).

**Figure 2:**
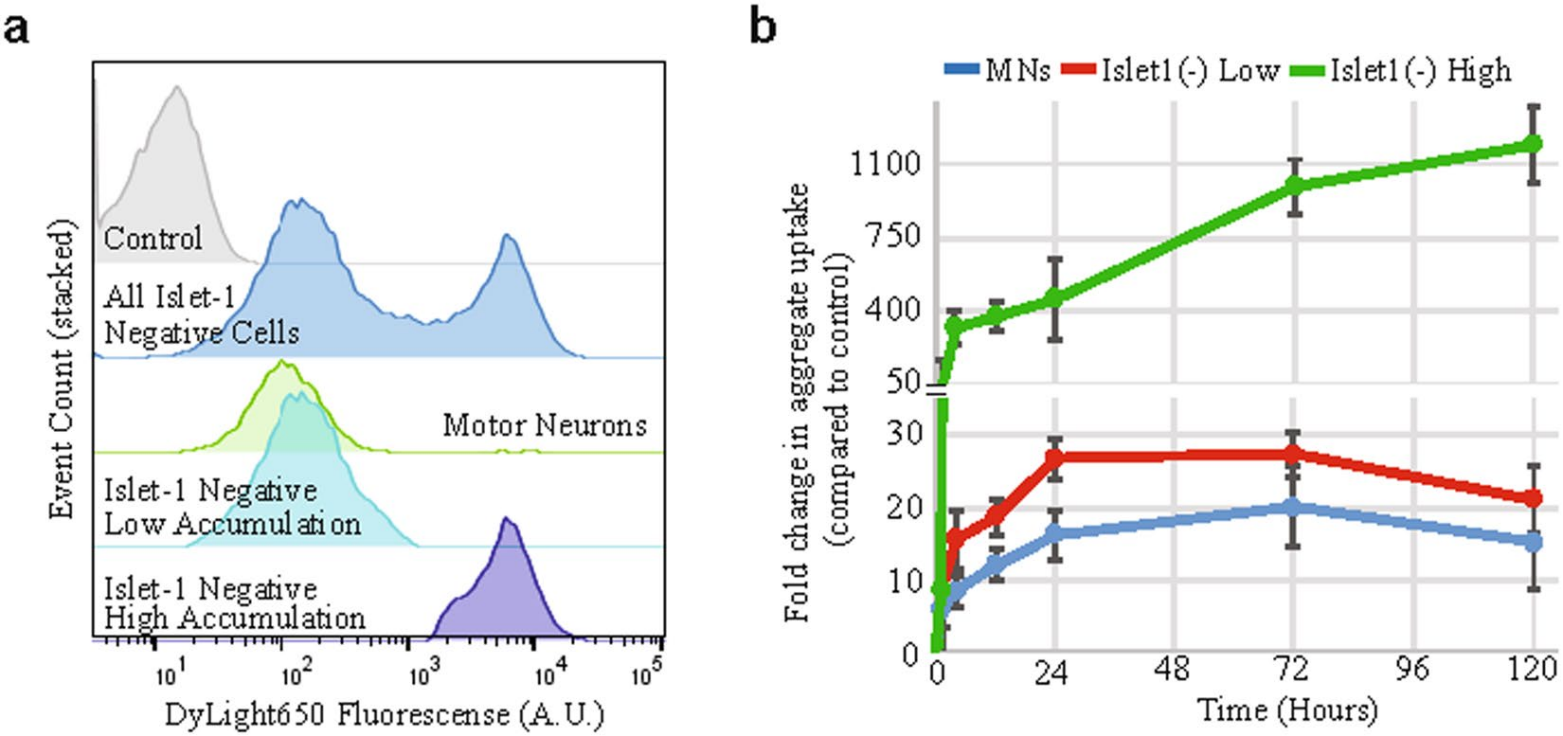
Rate of aggregate uptake by different cells in the mixed MN cultures. (**a**) A representative flow cytometry analysis showing that the MNs tend to accumulate relatively smaller amounts of aggregates while the Islet1(−) cells separate into two populations based on their accumulation of labeled SOD1^H46R^ aggregates. Cells were incubated with SOD1^H46R^ aggregates for 3 days prior to analysis. A.U, arbitrary units. (**b**) Quantification of FACS data over time of the accumulation of aggregates in MNs and the Islet1(−) populations. Fold change is calculated as the average intensity ± SD normalized to untreated control. A 2-way ANOVA shows that the samples (row factor) are significantly different (p = 0.0081). A multiple comparisons test shows that at 120 hours, the MN and Islet1(−) Low groups become significantly different from the Islet1(−) High group (p = 0.0197 and p = 0.0203, respectively). (n = 3).

Next we wished to establish whether SOD1 aggregates can be taken up by hPSC derived MNs and the other cell types in these cultures. We added the SOD1^H46R^ aggregates to MN cultures and incubated them for up to 5 days. Using FACS analysis to measure the amount of SOD1^H46R^ aggregate uptake (based on the DyLight 650 signal), we found that both the WT and SOD1^H46R^ aggregates were taken up by cultured cells in a time dependent manner (Fig. 1b, Supplementary Fig. S1b). To determine if these aggregates had truly been internalized and were not simply attached to the extracellular surface, we evaluated the aggregates’ sensitivity to trypsin digestion. When treated directly with trypsin, SOD1 aggregates are fully digested within 5 minutes. However, after incubation with the MNs, SOD1 aggregates are still present, suggesting they are internalized and thus protected from the trypsin digestion (Supplementary Fig. S1c and d). Consistent with this, using confocal microscopy, we could identify DyLight 650 SOD1 aggregates inside MNs (Fig. 1c, Supplementary Fig. S1e). To determine if aggregate uptake was restricted to MNs, we compared uptake in MNs to uptake in other cells in the culture (including neuronal and non-neuronal cells). Using FACS analysis and coupling labeled aggregate treatment with an Islet1-GFP motor neuron embryonic stem cell (ESC) reporter line^34^, we were able to determine the specific MN accumulation of DyLight 650 labeled SOD1^H46R^ aggregate uptake (GFP positive and DyLight 650 positive) and compare it to that of the Islet1 negative cells (GFP negative and DyLight 650 positive) within the same culture. We found that Islet1 negative cells could be separated into two distinct populations based on the level of accumulated SOD1 aggregates (Fig. 2a). We first observed the difference between the two populations 4 hours after the addition of the aggregates, with the difference becoming more pronounced over time (Fig. 2b, Supplementary Fig. S2a–c). After 72 hours, the strongly aggregate positive cells constituted 45 ± 13.3% of the total Islet1 negative cell population (quantification of Supplementary Fig. S2c). After 5 days, the highest accumulating cells had taken up 50-fold more aggregates than the other cell types in the same culture (Fig. 2b). Although similar, the lower accumulating Islet1 negative cells showed a consistent trend towards higher SOD1 aggregate accumulation over time compared to the Islet1 positive MNs (Fig. 2b).

### Mechanism of SOD1-Aggregate Uptake

**Figure 3:**
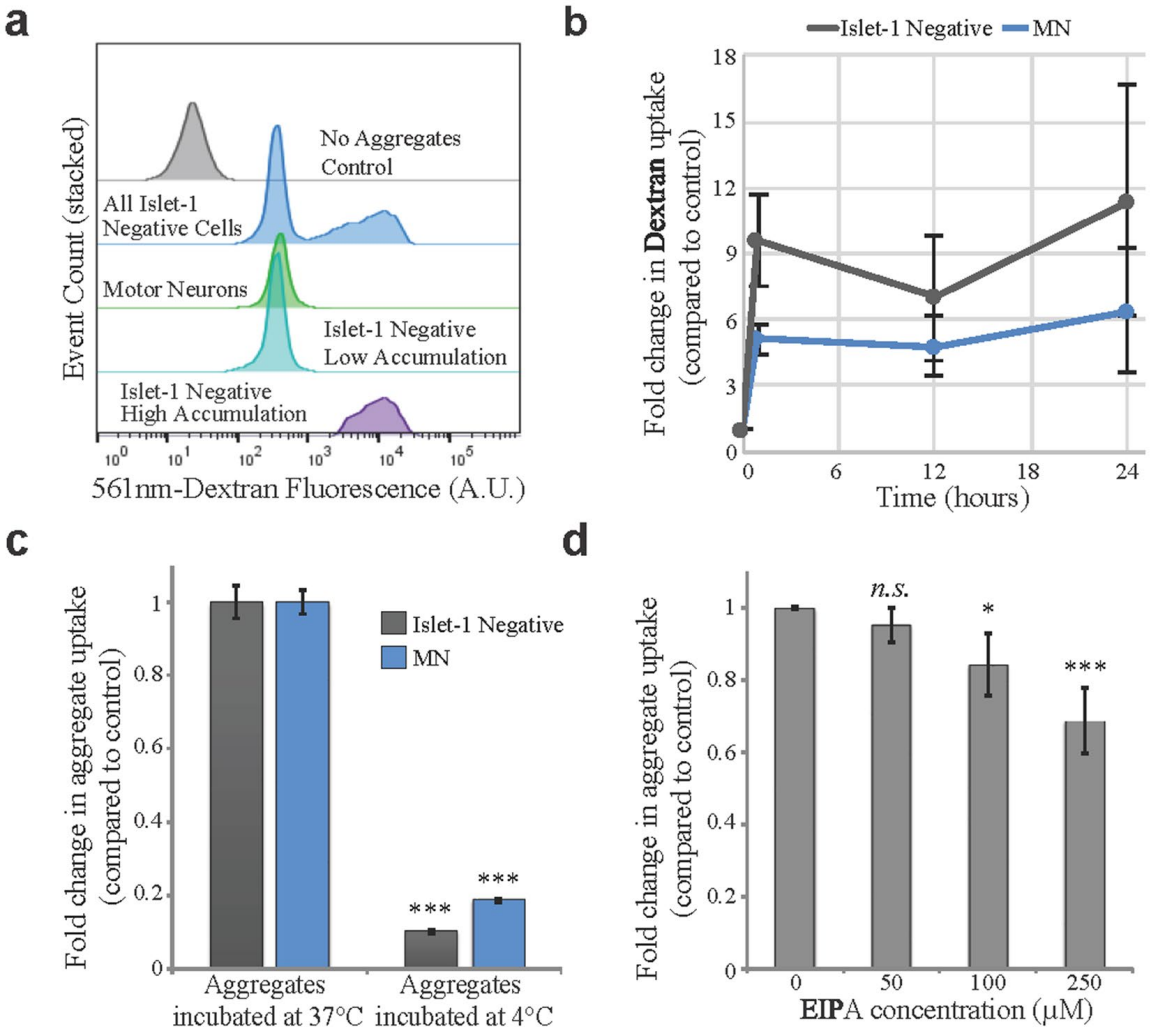
SOD1^H46R^ aggregate uptake and accumulation is temperature dependent and initially follows a similar rate as the fluid phase endocytosis marker dextran. (**a**) A representative flow cytometry analysis showing that dextran is taken up by all cells in the culture. The MNs and Islet1(−) cells separate into distinct groups by the amount of dextran accumulated after 24 hours. Dextran accumulation was determined by the intensity of its 561 nm fluorescent label. A.U., arbitrary units. (**b**) Quantification of the accumulation of dextran in the MN and Islet1(−) population over time. Fold change is calculated as the average intensity ± SD normalized to untreated control. A Friedman test shows the Islet1(−) and MN groups are slightly significantly different (p = 0.042). (**c**) Quantification of the reduction in SOD1^H46R^ aggregate uptake in response to low temperature in MNs compared to Islet1(−) cells. An unpaired two-tailed t test shows the 4 °C groups are highly significantly different from their 37 °C counterparts (p < 0.001). (n = 15) (**d**) Uptake inhibition by EIPA is dose dependent. Data are fold change in average intensity ± SD normalized to control. A repeated measures ANOVA shows that EIPA treatment significantly effects the uptake (p = 0.0016). A paired two tailed t test shows significant difference between the 100 µM and 250 µM groups and the control (0 µM) group (*p = 0.015, ***p < 0.001). (n = 9).

To elucidate how the SOD1^H46R^ aggregates entered the cells, we repeated the uptake study using Texas red-dextran (~70,000 MW) as a non-specific marker of fluid phase endocytosis. We found that, similar to the SOD1 aggregates, the culture separated into 3 distinct populations: the Islet1 positive MNs, and high and low accumulating Islet1 negative cells (Fig. 3a, Supplementary Fig. S3a). Again, the low accumulating Islet1 negative cells showed a consistent trend towards higher dextran over time when compared to the Islet1 positive MNs (Fig. 3b). While both SOD1 aggregates and dextran were initially quickly internalized, the SOD1 aggregates continuously accumulated in the cells over time while the dextran uptake plateaued (Fig. 2b compared to Fig. 3b and Supplementary Fig. S3b). This suggests that the SOD1 aggregates enter the cells in a non-specific process, similar to dextran, but are then processed differently and are not exocytosed or degraded with the same efficiency.

To further characterize the uptake process and to determine whether the SOD1^H46R^ aggregates entered the cells via an active process, we incubated the aggregates with the MNs at 4 °C or at 37 °C. We found that incubation at 4 °C almost completely abolished aggregate uptake (81.4 ± 0.4% reduction in MNs; 89.9 ± 0.3% in Islet1 negative cells, Fig. 3c and Supplementary Fig. S3c). Thus, SOD1 aggregates are taken up via an active, temperature-sensitive process.

Previous work using neuronal cell lines (N2A and NSC-34) suggested that SOD1 aggregate uptake is mediated by fluid phase endocytosis^27,28^. We sought to elucidate if the behavior of proliferating neuronal cell lines might differ from that of the terminally differentiated MNs affected in ALS. To test this, we evaluated which cellular uptake systems might be involved specifically in MNs. We first assessed the role of macropinocytosis using EIPA, IPA3 and wortmannin, known pharmacological inhibitors^28,35–40^. We found that all 3 macropinocytosis inhibitors significantly reduced the uptake in a dose dependent manner (Fig. 3d and Supplementary Figs S3d, S4a,b). We also evaluated additional pharmacological treatments known to block actin, dynamin, or caveolin dependent endocytosis along with treatments to block lipid raft or clathrin mediated endocytosis (Supplementary Fig. S4). Although some inhibitors gave statistically significant uptake reduction, no single inhibitor could block more than ~40% of the aggregate uptake (Supplementary Fig. S4). This leads us to conclude that uptake is not pathway specific but does involve fluid phase endocytosis. Thus, in terms of uptake by MNs, our results were in line with those observed in N2A and NSC-34^27,28^, re-enforcing the involvement of fluid phase endocytosis in SOD1 aggregate uptake.

### Toxicity of SOD1-Aggregates

**Figure 4:**
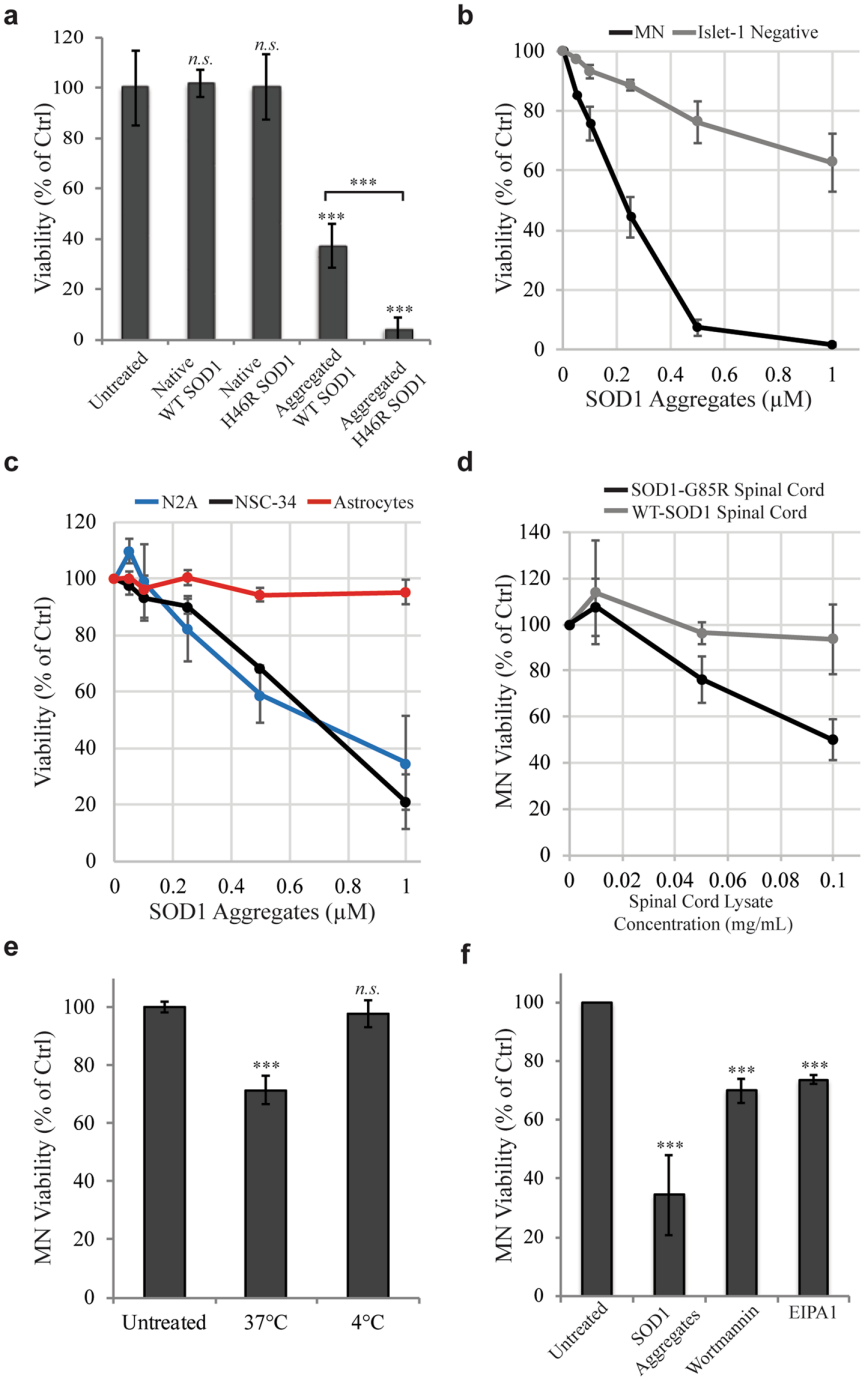
SOD1^H46R^ aggregates are selectively toxic to MNs and toxicity can be mitigated by aggregate uptake inhibition. (**a**) After 5 days of treatment, aggregated WT and H46R SOD1 are significantly more toxic to MNs than their native counterparts and mutant aggregates are more toxic than WT aggregates. Significance was calculated using an unpaired two tailed t test to compare each treatment individually to the untreated control, or between Aggregated WT SOD1 and Aggregated H46R SOD1 (denoted with brackets). n.s. = not significant, ***p < 0.001 (n = 12) **(b**) Aggregate sensitivity is dose dependent and MNs are more sensitive to SOD1^H46R^ aggregate induced toxicity than the Islet1(−) cells, which only respond at higher concentrations of aggregates (p = 0.032, repeated measures one-way ANOVA). Quantified after 5 days of treatment. (n = 4) (**c**) SOD1^H46R^ aggregate dose response of cell viability in the neuronal cell lines N2A and NSC-34 and human astrocytes showing that they are less sensitive than MNs (p = 0.0045, comparing MN (data in Fig. 4b) to N2A and NSC-34 using a repeated measures one-way ANOVA). Quantified after 5 days of treatment. (n = 4) (**d**) Spinal cord extracts from mice overexpressing human SOD1 G85R but not WT hSOD1 are toxic to MNs. p = 0.0011 using a two-way ANOVA. Quantified after 5 days of treatment. (**e**,**f**) SOD1^H46R^ aggregate toxicity in MNs can be mitigated by blocking aggregate uptake through incubation at 4 °C for 4 hours or by treatment with Wortmanin or EIPA1 for 6 hours. Significance was calculated using an unpaired two tailed t test to compare each treatment individually to the untreated control for the experiment (n.s. = not significant, ***p < 0.001). (**e** n = 14, **f** n = 6) Quantification of data are means ± SD normalized to control.

Next, we wanted to evaluate the potential toxic effects of the SOD1^H46R^ aggregates, with a particular focus on comparing MNs to other cell types. We found that *unaggregated* forms of WT and SOD1^H46R^ proteins were not toxic to the cultures, at least over the time periods used in these experiments (Fig. 4a). However, following aggregation, both were toxic (Fig. 4a). Despite being taken up and accumulating similarly (Fig. 1b), SOD1^H46R^ aggregates were significantly more toxic than WT-SOD1 aggregates after 5 days (Fig. 4a). We also found that low doses of the SOD1^H46R^ aggregates were significantly more toxic to MNs than to Islet1 negative cells within the same culture (EC50 for death being approximately 0.2 μM for motor neurons and >1 μM for the other cells (Fig. 4b)). The neuronal cell line N2A, as well as the motor neuron cell line NSC-34, readily took up SOD1 aggregates (Supplementary Fig. S1a), but were much more resistant to their toxic effects (Fig. 4c; EC50 approximately 0.7 μM). Effects on proliferating cells are likely to also include reduced proliferation following aggregate uptake, making the difference in sensitivity to toxic effects somewhat greater. Despite being in direct contact with MNs, astrocytes are relatively preserved in the progression of ALS. Interestingly, we found that human astrocytes readily took up and accumulated SOD1^H46R^ aggregates (Supplementary Fig. S1a); yet, they were almost entirely resistant to their toxic effects even at high concentrations (Fig. 4c). For an additional control, we also evaluated the effects of aggregated DyLight 650 labeled BSA aggregates, which proved to be not toxic to any of the cell types measured (Supplementary Fig. S5a). Taken together, these results suggest a selective MN effect that occurs downstream from aggregate uptake.

To further validate our *in vitro* model, we sought to evaluate the toxic effect of SOD1 aggregates that were isolated from the spinal cords of ALS mice. To this end, we used the soluble fraction of spinal cord homogenates prepared from mice over-expressing either the native human *SOD1* gene or the *SOD1*^G85R^ gene^21^. We found that preparations containing the G85R mutated SOD1 were toxic to MNs, whereas their WT counterparts were not (Fig. 4d). To further confirm the direct involvement of the SOD1 protein in this toxic process, we depleted it from the spinal cord preparation using anti-SOD1 antibodies. We were not able to obtain 100% depletion (perhaps due to the aggregation of the protein) but nonetheless saw a significant reduction in toxicity of about 20% (Supplementary Fig. S5b,c).

To further study the connection between SOD1 aggregate uptake and cellular toxicity, we selected 3 conditions that efficiently reduced aggregate uptake in this study (reduced temperature and exposure to wortmannin or EIPA) and examined aggregate toxicity under these treatments. We did not include IPA3 in this study as it proved too toxic and would confound viability measurements. We found that all of these treatments significantly improved the MN viability, indicating a correlation between uptake inhibition and reduced toxicity (Fig. 4e,f).

### Downstream Effects of SOD1 Aggregate Uptake

To evaluate the cellular responses to SOD1^H46R^ aggregate treatment, mixed MN cultures produced from the Islet1-GFP reporter line were incubated with SOD1^H46R^ aggregates for 5 days. The culture was then FACS sorted into MN (Islet1-GFP^+^) and non-MN (Islet1-GFP^−^) pools. They were then analyzed by qPCR for changes in gene expression compared to their respective untreated groups. Because aggregates are misfolded proteins, we chose to examine known cellular pathways that respond to protein misfolding.

**Figure 5:**
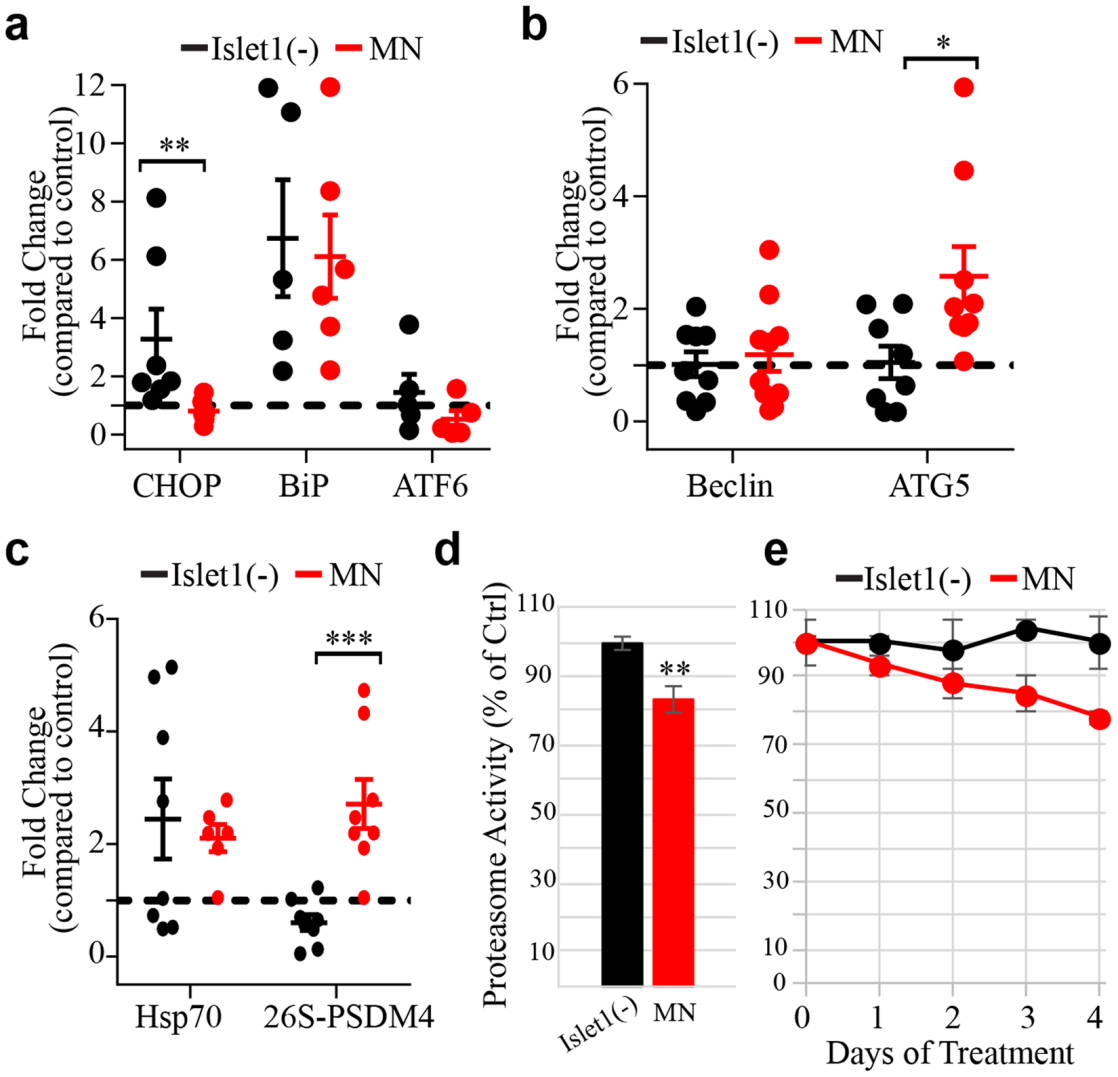
qPCR analysis of proteastasis markers in SOD1^H46R^ aggregate treated MNs versus Islet1(−) cells from the same culture. (**a)** ER stress markers *CHOP*, *BiP*, and *ATF6* are stimulated in the Islet1(−) population while the MNs only increase expression of *BiP* after treatment with SOD1 aggregates. (**b**) The autophagy genes *Beclin* and *ATG5* were not stimulated in the Islet1(−) group while *ATG5* expression was increased in aggregate treated MNs. (**c**) *Hsp70* transcription was stimulated by aggregate treatment in both groups. The regulatory subunit of the 26 S proteasome, *PSDM4*, was specifically activated in treated MNs. (**a**–**c**) Each point indicates the average of two technical replicates from one biological replicate. Each bar indicates the average of biological replicates ± SEM. Significance was calculated using a Mann-Whitney test, *p ≤ 0.05, **p ≤ 0.01, ***p ≤ 0.001. (**d**) Proteasome activity in FACS purified untreated Islet1(−) cells (normalized to 100%) to untreated MNs. Activity is significantly less in the MNs (calculated with an unpaired two-tailed t test, **p = 0.0048). (**e**) The cell types were normalized to their own untreated control, either MN or Islet1(−), and the data represents percent proteasome activity, as compared to their respective untreated control, over a SOD^H46R^ aggregate treatment time course. Using a repeated measures one-way ANOVA, the difference in the treated MN compared to treated Islet1(−) normalized time course data is significant (*p = 0.046).

We first looked at the endoplasmic reticulum (ER)-stress pathway via its canonical stress genes *CHOP*, *BiP* and *ATF6*. Islet1-negative cells that were aggregate treated showed an up-regulation of *CHOP* and *BiP* compared to the untreated Islet1-negative cells, whereas aggregate treated MNs only showed upregulation of *BiP* (Fig. 5a). Next, we investigated whether macroautophagy may be involved in responding to aggregate treatment through the canonical genes *Beclin* and *ATG5*. Compared to their untreated counterparts, aggregate treated Islet1-negative cells did not change their expression of these genes while aggregate treated MNs increased expression of only *ATG5* (Fig. 5b). Lastly, we evaluated the unfolded protein response through the expression of the chaperone *Hsp70* and the 26S proteasome. Both Islet1-negative cells and MNs treated with aggregates had increased transcription of *Hsp70* (Fig. 5c). Interestingly, *PSDM4*, a regulatory subunit of the 26S proteasome, was found to be up-regulated only in aggregate treated MNs (Fig. 5c). Using a Mann-Whitney test, *Chop, ATG5*, and *PSDM4* were all significantly differentially regulated between the treated MNs and the treated Islet1-negative cells.

To confirm that the proteasome is affected by SOD1^H46R^ aggregate treatment, we performed a proteasome activity assay on FACS purified MNs compared to Islet1-negative cells from the same culture. Comparing untreated MNs to untreated Islet1-negative cells, the MNs already have a significant reduction in proteasomal activity (83% of the Islet1-negative activity, Fig. 5d). When treated with aggregates for 1, 2, 3, or 4 days, the Islet1-negative cells did not lose any proteasomal activity compared to their untreated control. However, the MNs continued to lose activity overtime; down to 78% of the activity of untreated MNs by day 4 (Fig. 5e). The mismatch of upregulation in the transcription of the proteasome, but a decrease in activity, suggests that the upregulation is compensating for the inefficiency of potentially aggregate-blocked proteasomes, and the efficiency *per proteasome* is most likely lower than 78% in treated MNs.

### Effects of SOD1 Antibodies on Aggregate Uptake and Toxicity

**Figure 6:**
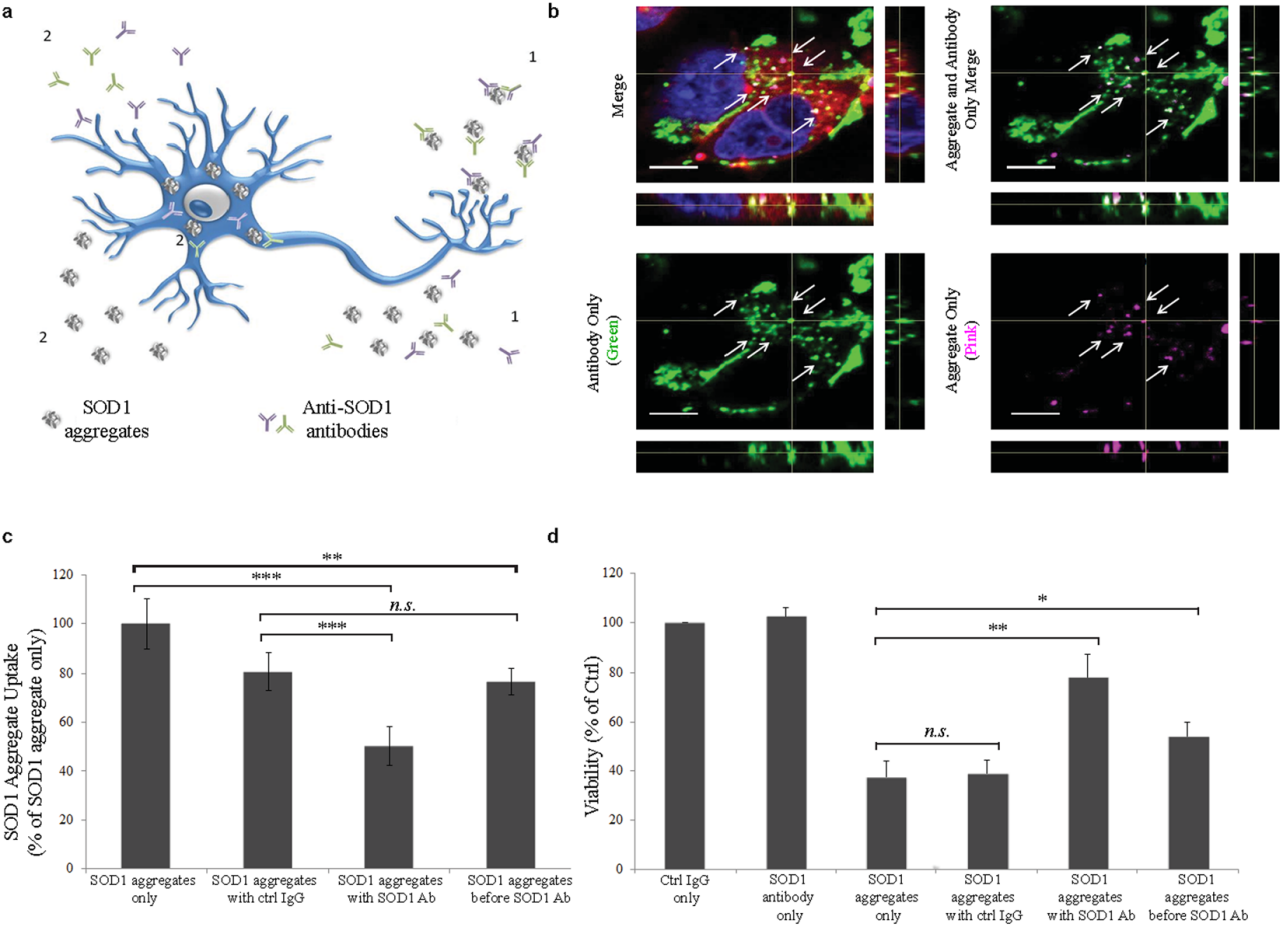
Inhibition of aggregate uptake and toxicity through treatment with antibodies against SOD1. (**a**) A schematic representation of the potential pathways by which αSOD1 antibodies might reduce the SOD1^H46R^ aggregate toxicity: 1. Reduce uptake by binding aggregates outside the cell. 2. An uptake independent pathway where antibodies interact with aggregates inside the cell. (**b**) Representative confocal image illustrating that αSOD1 antibodies (labeled with Alexa488, green) can be found inside cells associated with SOD1^H46R^ aggregates (magenta). Overlap indicated in yellow and by white arrows. The lipophilic dye FM 4–64FX (red) marks the cell periphery. Scale bar = 5 µm. (**c**) SOD1^H46R^ aggregate uptake in MNs under different aggregate:antibody treatments after 5 days. Incubation of SOD1^H46R^ aggregates with αSOD1 antibodies before addition to cells significantly reduces aggregate uptake, whereas incubation with control antibodies as well as incubation with αSOD1 antibodies after the aggregates have been internalized (sequential addition) have less effect on SOD1^H46R^ uptake. (n = 9) (**d**) Viability of MNs under different aggregate:antibody treatments after 5 days. Pretreatment of SOD1^H46R^ aggregates with antibody, as well as sequential antibody treatment, reduces aggregate toxicity. (n = 9) Quantification of data are means ± SD normalized to control. Significance was calculated using an upaired two tailed t test comparing each treatment individually to their respective control (denoted with brackets). n.s. = not significant, *p ≤ 0.05, **p ≤ 0.01, ***p ≤ 0.001.

There has been a great deal of clinical interest in using antibodies to block the spread of aggregated proteins as a novel type of treatment for neurodegenerative disease (Fig. 6a). This led us to investigate the effects of a polyclonal rabbit anti-SOD1 antibody in our ALS model in two ways: through the addition of aggregates and antibodies together or by allowing aggregates to first interact with the cell and then add the antibodies (sequential addition).

We first incubated the SOD1^H46R^ aggregates with the SOD1 antibody for 1 hour and then added the aggregate-antibody solution to the MN cultures and allowed them to incubate for 5 days. Under these conditions, we found SOD1 antibodies associated with the SOD1 ^H46R^ aggregates inside the MNs as well as in the extracellular space (Fig. 6b,c, Supplementary Fig. S6a–d).

In a separate experiment, MNs were first exposed to the SOD1^H46R^ aggregates for 12 hours and then washed to remove free SOD1 aggregates from the media. Immediately thereafter, SOD1 antibodies were added to the cells and the cultures were kept for 4.5 more days. Under these sequential addition conditions, we again detected antibody-aggregate association inside the cells as well as outside (Fig. 6c, Supplementary Fig. S6e–h). Interestingly, under the sequential addition condition, we observed fewer large, coalesced aggregates (aggregates of aggregates) inside the motor neurons (Supplementary Fig. S6). We also observed that the fluorescently labeled antibodies, when added under the sequential addition condition, were found outside cell membranes (Supplementary Fig. S6g).

To understand if antibodies themselves can enter MNs, we incubated MNs with a dose curve of fluorescently labeled polyclonal rabbit antibodies (rabbit-Alexa568, final concentration range of 1.25 µg/mL–40 µg/mL) for 3 days. The cells were then dissociated with trypsin to remove antibodies associated with the cell surface and analyzed with FACS. Results show a clear and dose-dependent increase in antibody-Alex568 fluorescence in MNs, confirming that even antibodies in suspension are taken up by MNs within 3 days (Supplementary Fig. S7).

To quantify these observations, we evaluated the effect of antibody addition on SOD1 aggregate uptake and toxicity. Using FACS to measure the DyLight 650 SOD1^H46R^ aggregate concentration inside cells, we found that the SOD1 antibody reduced the uptake levels significantly when compared to the control antibody (49.7% compared to 19.4% reduction, Fig. 6c). When the SOD1 antibodies were added to the cells after the SOD1^H46R^ aggregates had been removed from the medium, intracellular levels of aggregates were also significantly reduced (Fig. 6c).

To evaluate the effects of the SOD1 antibodies on aggregate toxicity, we treated the MN cultures as before with SOD1^H46R^ aggregates in the presence or absence of SOD1 antibodies. We found that treatment with antibody during the aggregate exposure significantly reduced the toxicity of SOD1^H46R^ aggregates, potentially due to reduced aggregate uptake (the MN viability increased by 2.1-fold as a result of antibody treatment, Fig. 6d). Furthermore, when MN cultures were treated with the antibodies after the SOD1^H46R^ aggregates were removed, we still observed a 1.4-fold increase in survival, providing a significant, albeit reduced, neuroprotective effect even though aggregate uptake was not reduced compared to the control IgG (Fig. 6d).

## Discussion

In this report, we have shown that motor neurons are specifically vulnerable to SOD1 aggregate induced toxicity. Despite extensive efforts, the underlying causes of many neurodegenerative diseases, including ALS, remain elusive. Numerous cellular and animal models have been developed, but have not translated into effective therapies for patients^41–44^. The late onset, disease complexity, as well as the difficulty in obtaining human MN samples from affected individuals, all contribute to the uncertainty of the causes of ALS and of potential treatments. The use of human pluripotent cells has many advantages in neurodegenerative disease research. This new technology provides the possibility of patient-specific therapy and also provides a well-defined system for studying the development of ALS in the cell types most affected in the disease.

For these reasons, we chose to use human pluripotent cells differentiated into MNs to elucidate the involvement of SOD1 aggregates in the pathophysiology of ALS. Using MNs, we evaluated the cell specific toxicity of SOD1 aggregates, made with either WT SOD1 or a familial ALS causing mutant form of SOD1 (H46R), and compared these results to those obtained from the neuronal cell lines used previously^27,28^. We have confirmed that SOD1 aggregate uptake into MNs and other cells in stem cell-derived cultures is an active endocytotic process^27,28^. Importantly, we also found that this endocytotic process is not cell type specific and that numerous cell types are capable of internalizing SOD1 aggregates. Our results are also supported by recent *in vivo* observations demonstrating that inside the spinal cord, SOD1 aggregates are progressively and stably formed as well as transported to neighboring cells within the spinal cord. These studies found that MN to MN spread may be assisted by oligodendrocytes^21^. Importantly, in our study, we found that while multiple types of cells can take up aggregates, MNs are the most adversely affected by them. Thus, toxicity is not related to absolute amount of uptake.

Interestingly, we found that non-MNs readily take up and accumulate aggregates, but survive well even after 5 days of treatment. These cells mount a modest ER stress response, activate *Hsp70*, and maintain their proteasome activity. This modest response and the lack of proteasome activation suggests that the ER stress response in the non-MNs may support their survival. In MNs, it is known that the formation of endogenous intracellular aggregates activates the ER stress pathway and ultimately leads to death^45^. Surprisingly in MNs that were treated with exogenously added aggregates and survived for 5 days, no ER stress response was observed. Instead, these cells appeared to increase proteasomal and, perhaps, macroautophagic, degradation. Even though the expression of proteasome is increased in aggregate treated MNs, we found that the activity of the proteasome was diminished in a time dependent manner. This suggests that while endogenously formed aggregates cause an ER stress response, aggregates that are internalized and accumulated from the extracellular space bypass this protective response. Our results indicate that protein degradation pathways are instead used to cope with internalized aggregates and can be neuroprotective for some amount of time. In fact, reduced proteasome activity has been measured in the spinal cords of SOD1 mutant mice compared to their WT counterparts^46^.

Antibodies against proteins that aggregate in neurodegenerative diseases, such as Alzheimer’s and Parkinson’s, are being developed for clinical use and have even shown potential in ALS cellular and mouse models^27,47–52^. It is thought that the antibodies reduce the cell-to-cell spread of the aggregates as well as increase the clearance of the aggregates by neighboring cells. Here we confirm that SOD1 antibodies reduce the uptake of SOD1 aggregates, thus conferring some protective potential. However, we also found that antibodies added after cells had internalized aggregated SOD1 continued to have some beneficial effect. Possibly, the antibodies themselves are internalized over time (as other proteins are and as we show in Supplementary Fig. S7), bind to the aggregates, and either target them for degradation or otherwise reduce their ability to cause cell death. Alternatively, the antibodies could remain extracellular and prevent the re-uptake of released aggregates. In this study, the concentration of antibodies used was significantly higher than that likely to be achieved with a systemically administered therapeutic antibody. Nonetheless, these experiments suggest that the toxicity of intracellular aggregates can still be reduced by a therapeutic that interferes with their uptake or even with the effects of internalized aggregates. The latter effect might be more readily achieved with a cell permeable small molecule.

In conclusion, these findings highlight the importance of using disease relevant cells to understand the entire cascade of events from the uptake of aggregated disease-causing proteins, to their eventual spread and death of the “host” cell. While some aspects of this process can be studied using neurons or even other cell types, at least one of the most important steps – cell death – seems best studied with the type of cells most affected by the disease-causing protein aggregate. We therefore believe that these types of *in vitro* models recapitulate more closely the disease and, therefore, will make important contributions to the identification and evaluation of potential therapeutics.

## Methods

### Human pluripotent cell lines

The use of the iPSC and ESC lines was reviewed by the Harvard Committee on the Use of Human Subjects (the Harvard IRB) and determined to not constitute human subjects research.

### Human pluripotent cell culture and differentiation

Human iPSCs and ESCs were maintained and differentiated as previously described (Rigamonti, *et al*.^33^) briefly; cells were maintained in mTeSR1 (05850, STEMCELL Technologies) and then adapted to 125-ml disposable spinner flasks (89089, VWR) and mixed at a speed of 55 rpm, in a 37 °C incubator with 5% CO_2_. Prior to adaption to spinner-flask culture, cells were expanded in 15-cm dishes and dissociated to single cells with Accutase (07920, STEMCELL Technologies). 50 million individual pluripotent stem cells were seeded into a spinner flask at a concentration of 1million cells/mL in mTeSR media supplemented with 5 μM ROCK inhibitor Y-27632 (688000, EMD Millipore). Spheres formed spontaneously, and after 2 days the culture medium was changed. At day 1 of differentiation, medium was changed to mTeSR with the activin/TGF-β inhibitor SB431542 (bulk custom synthesis, R&D Systems) (10 μM) and the BMP inhibitor LDN193189 (04-0074-02, Stemgent) (1 μM) (hereafter referred to as “dual SMAD inhibition”). From days 2 to 10, spheres were gradually adapted to NIM [neural induction medium (NIM): DMEM/F12 (11330057, Life Technologies), 1× N2 supplement (175022048, Life Technologies), 1× B27 supplement (17504044, Life Technologies) 1× Glutamax (35050061, Life Technologies), 1× non-essential amino acids (NEAA, TMS-001-C, EMD Millipore), 1× penicillin-streptomycin (15140163, Life Technologies), 3.2 mg/ml glucose (15023, Gibco), and 0.2 mM ascorbic acid (A4403, Sigma Aldrich)] through a dilution series of KSR [KSR medium: 15% KOSR (10828028, Life Technologies), KO DMEM (10829018, Life Technologies), 1× Glutamax, 1× NEAA, 1× penicillin-streptomycin, and 1× β-mercaptoethanol (21985023, Life Technologies)] and NIM, with dual SMAD inhibition maintained until day 6. Medium was changed as follows: day 2: 100% KSR; day 3: 100% KSR +1 μM retinoic acid (RA) (R2623, Sigma Aldrich); day 5: 75% KSR, 25% NIM + RA, and 10 ng/ml BDNF (248-BD-01M, R&D); day 6: 50% KSR, 50% NIM + RA, 1 μM Smoothened agonist (SAG) (custom bulk synthesis, Curis), and BDNF; day 8: 25% KSR, 75% NIM + RA, SAG, and BDNF. From days 10 to 15, cultures were maintained in 100% NIM + RA, SAG, BDNF, and 2.5 μM DAPT (2634, R&D). On day 15 the spheres were dissociated with Accutase and gentle mechanical agitation. Cells were then washed twice in NB medium, counted, resuspended in NB media [Neurobasal (NB) medium: Neurobasal (21103049, Life Technologies), 1× N2 supplement, 1× B27 supplement, 1× Glutamax, 1× NEAA, 1× penicillin-streptomycin, 3.2 mg/ml glucose and 0.2 mM ascorbic acid]. The medium was supplemented with 10 ng/ml brain-derived neurotrophic factor (BDNF) and 10 ng/ml glial cell-derived neurotrophic factor (GDNF, 512-gf-010, R&D Systems) and 10 ng/ml ciliary neurotrophic factor (CNFT, 557-NT, R&D systems), filtered and plated in plates coated with laminin (0.025 mg/mL, 23017015, Life Technologies), Poly-L-ornithine hydrobromide (0.025 mg/mL, 62405-436, VWR), poly-D-lysine (0.5 mg/mL, A-003-E, EMD Millipore) and Fibronectin (0.01 mg/ml, 47743-654, VWR). The cells were maintained in NB media supplemented with BDNF, GDNF and CNTF.

### Culture of cell lines (NSC-34, N2A and human astrocytes)

Human astrocytes were purchased from ScienCell Research Laboratories (1820). The cells were maintained in DMEM (11330057, Life Technologies) containing 10% (v/v) FBS (F2442, Sigma Aldrich) and supplemented with 2 mM L-glutamine, 1× glucose, 1× penicillin-streptomycin at 37 °C with 5% CO_2_. The cells were passaged at 80% confluency with trypsin twice weekly.

### Immunohistochemistry

Cell cultures were fixed in 4% paraformaldehyde (100503, VWR) in PBS at room temperature (RT) for 20 min, then blocked for 1 hour in 10% normal goat serum (GS) (G9023, Sigma Aldrich) and 0.1% Triton X-100 (X100, Sigma Aldrich) in PBS. Cells were incubated with primary antibodies for 3 hours at RT. Secondary antibodies were applied for 1 hour in blocking buffer at RT. The primary antibodies used in this study were raised against rabbit ISL1 (ab109516, Abcam), rabitt-anti-Ki67 (ab15580, Abcam), rabbit-anti-GABA (A2052, Sigma Aldrich), mouse-anti-SMI32 (801701, BioLegend), rabbit-anti-MAP2 (AB5622, EMD Millipore), rabbit-anti-TUJ-1 (MRB-435P, BioLegend) Secondary antibodies were donkey and goat AlexaFluor488, 546, 555, and 650-conjugated antibodies raised against the appropriate species (used at 1:1000, Invitrogen). Where stated, the lipophilic membrane dye (FM 4-64FX, F34653, Molecular Probes) was used following the manufacturer’s instructions. Immunocytochemical images were acquired with an automated microscope (PerkinElmer Operetta), quantitative image analysis was performed using Columbus software (PerkinElmer) and Harmony High-Content Imaging software (PerkinElmer). Confocal images were acquired with a LSM 700 Inverted Confocal Microscope.

### Purification, labeling, and aggregation of WT SOD1 and SOD1^H46R^

Human erythrocyte-derived SOD1 protein (WT SOD1) was purchased from Sigma (catalog no. S9636), resuspended following the manufacturer’s instructions, and then subjected to size exclusion chromatography (SEC, Superdex200 16/60) to remove the high level of endotoxin in the material. After SEC, endotoxin levels were measured using an Endosafe-PTS instrument (Charles River) following the manufacturer’s protocol and confirmed to be below 0.5 EU/mL.

SOD1^H46R^ was produced via an inducible vector (provided by Biogen, Idec.) in E. coli resulting in a protein with a GST-tag. The pelleted E. coli was lysed with freeze thaw cycles in lysis buffer [4 mL/gram of cell pellet, 50 mM Tris, pH 8, 400 mM NaCl, 1 mM EDTA, 1% Triton X-100, 3 mM DTT, 25 µg/µL DNase (LK003172, Worthington) and protease inhibitor (PI78426, VWR)] and then clarified via centrifugation. The crude lysate was applied to GST resin (5 mL per L of culture, 95055-026, GenScript) over night at 4 °C with end over end mixing. The slurry was then poured into a disposable chromatography column and washed with 20 column volumes of lysis buffer. Bound protein was eluted with competitive binding of 10 mM glutathione and the protein concentration was determined using UV/Visible spectroscopy (E^mM^ = 18.4, 265 nm). To remove the GST tag, PreScission Protease (95017-584, VWR) was added following the manufacturer’s directions, and the protein/enzyme mixture was dialyzed against GST wash buffer (50 mM Tris, pH 8, 400 mM NaCl, 1 mM EDTA, 3 mM DTT) at 4 °C over night. The next day, the dialysis buffer was changed to fresh GST wash buffer and continued for an additional 4 hours. The protein was applied to a clean GST column to remove the cleaved GST tag from the sample. Standard SDS-PAGE/Coomassie was run to confirm quantitative cleavage and purity.

Next, the protein was completely demetallized with extensive dialysis in Buffer A (50 mM Tris, pH 8, 200 mM NaCl, 100 mM EDTA) followed by Buffer B (100 mM Sodium Acetate), and then PBS. To remetallize the protein, 10 mM ZnCl_2_ (final concentration) was added directly to the protein and incubated overnight at 4 °C. Next, 10 mM CuSO_4_ (final concentration) was added directly to the protein and incubated overnight at 4 °C. The protein was dialyzed extensively in PBS, concentrated and applied to SEC as described above.

NHS-ester chemistry was used to label either the WT or SOD^H46R^ protein with a DyLight 650^TM^ molecule following the manufacturer’s protocol (62265, Thermo Fisher Scientific). Aggregates were generated by diluting to 10 mM in PBS and 20% (final concentration) 2,2,2-trifluoroethanol and incubating at room temperature, in the dark, overnight. Aggregates were aliquoted and stored at −80 °C.

### Flow cytometry

For flow cytometry experiments, the Islet1-GFP motor neuron reporter line was used^34^. For all experiements, cells were incubated with 0.2 µM (monomer equivalent) of DyLight 650 labeled WT or SOD1^H46R^ aggregates for the indicated times. Unless otherwise stated, before the analysis, the remaining inoculum was digested with 0.25% trypsin for 5 minutes, and the cells were collected for analysis by flow cytometry. Flow cytometry data capture and cell sorting were performed on a Mo-Flo cell sorter (Beckman Colter). Analysis was performed with the FlowJo software (Flowjo LLC.). Where indicated the DyLight 650 labeled WT or SOD1^H46R^ aggregates were replaced with the fluid phase endocytosis marker dextran (~70,000 MW, D1830, ThermoFisher), a small hydrophilic polysaccharide, at a concentration of 2.5 mg/mL.

### Perturbation of endocytosis

Unless otherwise stated, the cells were exposed to the specified inhibitor for 30 minutes before the DyLight650 labeled SOD1^H46R^ aggregates were introduced to the system, followed by 4 hours of concurrent treatment with the specified inhibitor as well as 0.2 µM (monomer equivalent) of DyLight 650 labeled SOD1^H46R^ aggregates. The inhibitors were used at the following concentrations: 50–250 µM of 5-(n-ethyl-n-isopropyl) amiloride (EIPA), 100 µM inhibitor targeting P21-activated kinase activation 3 (IPA3), and 50 nM Wortmannin; the actin-polymerization inhibitors cytochalasin A (20 µM) and D (50 µg/ml); 10 mM methyl-β-cyclodextrin (MβCD), 250 µM Dynasore, 100 µM Genistein, 40 µg/ml Nystatin, 50 µM 3-(2,4-Dichloro-5-methoxyphenyl)-2,3-dihydro-2-thioxo-4(1 H)-quinazolinone (Mdivi-1), 10 µM chlorpromazine (CHPZ), and 20 µM phenylarsine oxide (PAO). After inhibitor treatment, the cells were collected for analysis by flow cytometry. Cells were disrupted to single cells using Accutase and treated with 0.25% trypsin to assure all labeled aggregates on the cell surface were digested. Flow cytometry data capture and cell sorting were performed on a Mo-Flo cell sorter. Analysis was performed with the with FlowJo software.

### Toxicity, toxicity mitigation and survival

Unless otherwise stated, the cells were exposed to the specified concentration of the native or aggregated forms of WT or SOD1^H46R^, or aggregated BSA for 5 days. The viability was calculated based on fold change in the number of total live nuclei (stained with Hoechst H33259 dye) in the treated culture compared to an untreated control. The quantification of MNs was calculated based on fold change in the number of total live Islet-1 positive cells compared to an untreated control. Images were acquired with an automated microscope. Quantitative image analysis was performed using Columbus software and Harmony High-Content Imaging software.

To evaluate the effect of decreased SOD1^H46R^ aggregate uptake on cellular toxicity, the cells were exposed to several different conditions. Even at the relatively low concentrations of 100 µM EIPA and 50 nM Wortmannin, the cells could only sustain up to 6.5 hours of exposure to the inhibitors and 4 hours of exposure to 4 °C. Therefore, for the reduced temperature experiments, the cells were primed with 30 min of exposure to 4 °C, EIPA or Wortmannin, and then the SOD1^H46R^ aggregates were introduced to the cells and incubated at 4 °C for an additional 3.5 hours *or* incubated with EIPA or Wortmannin during the SOD1 aggregate exposure for 6 hours. The SOD1^H46R^ aggregates were then removed and the cells were washed twice with warm rich MN media. The cells were then kept in rich MN media for a total of 5 days from the moment the SOD1 aggregates were introduced to allow for the toxicity to slowly develop. The survival was evaluated using the aforementioned techniques.

### SOD1^H46R^ aggregate uptake reduction and toxicity mitigation using anti-SOD1 antibodies

The SOD1^H46R^ aggregates were incubated with a rabbit polyclonal anti-SOD1 antibody (ADI-SOD, Enzo Life Sciences) or a rabbit polyclonal IgG control (30000-0-AP, ProteinTech) for 1 hour at 4 °C. The cells were then incubated with the mixture of SOD1^H46R^ aggregates and antibodies in MN medium for 12 hours. For the SOD1^H46R^ aggregate control and the experiments where the SOD1^H46R^ aggregates and antibodies were incubated sequentially, the cells were incubated with SOD1^H46R^ aggregates for only 12 hours. At this point, the SOD1^H46R^ aggregates were removed and the cells were washed twice with warm rich MN media.

For the experiments where the SOD1^H46R^ aggregates and antibodies were incubated sequentially, the cells were first incubated with anti-SOD1 or normal IgG control antibodies in rich MN media for 12 hours. Cells from all conditions were washed twice with warm MN medium. Next, SOD1^H46R^ aggregates were added and the cells were kept in MN medium for a total of 5 days. The viability was calculated based on fold change in the number of total live nuclei (stained with Hoechst H33259 dye) compared to an untreated control while the MN viability was calculated based on fold change in the number of total live Islet-1 positive cells compared to an untreated control. Images were acquired with an automated microscope and analyzed as above. For uptake inhibition, the cells were collected at the end of 5 days for analysis by flow cytometry as described above. Flow cytometry data capture and cell sorting were performed on a Mo-Flo cell sorter. Analysis was performed with the FlowJo software. Confocal images were acquired with a LSM 700 Inverted Confocal Microscope.

### Transcriptional analysis via qPCR

Collected cell pellets were lysed with Trizol and RNA was extracted using chloroform and isopropanol. The RNA samples were first treated with DNase (18068-015, Invitrogen) before further use. 1 µg of RNA from each sample was reverse transcribed to cDNA using iScript (170-8890, Bio-Rad) following the manufacturer’s protocol. qPCR was carried out in technical duplicate using Fast SYBR Green (4385610, Applied Biosystems) and run on a Quant Studio 12k Flex instrument which calculated CT values and melt curves for each sample. Each sample had a single peak melt curve to assure quality of the primers and samples. Fold changes were computed from the average of the technical duplicates using Microsoft Excel. qPCR plots were generated using GraphPad Prism showing the average of many biological replicates and the SEM. A table of qPCR primers and their sequence can be found in the supplementary information.

### SOD1^H46R^ digestion with trypsin

1 µl of SOD^H46R^ aggregates (10 µM) were directly added to 20 µL of 0.25% trypsin and allowed to incubate at 37 °C for 5, 10, 15, and 20 minutes. When the time point was up, 5x protease inhibitors were added to the solution and it was placed on ice. Samples were then boiled for 5 minutes under reducing conditions and loaded on to a 10% hand-poured acrylamide gel. Once the bromophenol blue had exited the gel, the gels were washed in water and scanned on a Typhoon fluorescent gel scanner (Amersham Biosciences) using the Cy5 excitation/emission filter (voltage setting: 650 PMT) to excite the DyLight 650 labeled aggregates.

### SOD1^H46R^ Western Blot

Samples were boiled for 5 minutes under reducing conditions and loaded on to a prepared 4–15% acrylamide gel (4561085, Bio-Rad). The gel was then blotted on to a PVDF membrane (1704156, Bio-Rad) using a Bio-Rad semi-dry transfer unit. The membrane was blocked with 5% milk in PBST for 1 hour at room temperature and then probed with anti-SOD (1:2000) in 2% BSA overnight at 4 °C. The next day the membrane was washed 3 times in PBST, and probed with a goat-anti-rabbit-HRP (1:5000, PI31460, VWR) for 1 hour at room temperature. The membrane was then washed 3 times in PBST and imaged using a chemiluminescent substrate (Pierce catalog no. 34076) on a Bio-Rad gel imager.

### Purification of WT and G85R SOD1 spinal cords and SOD1 depletion

The full and purified spinal cord preparations were a kind gift from the Horwich Lab (Howard Hughes Medical Institute, Yale University School of Medicine). The spinal cords were obtained from WT and G85R-SOD1-YFP mouse strains and purified using Anti-YFP Protein A UltraLink resin as previously described. (Hadzipasic, *et al*. 2014)

### Statistics

Standard error and standard deviation were calculated using Microsoft Xcel and GraphPad Prism software. Statistical test were performed in GraphPad Prism 7.03. For all significance, the confidence interval was set to 95%. Due to our statisitcally low (less than 20) replicate size, we chose to use parametric tests in experiments were the standard deviation was low and comparable between samples. However when there was considerable spread in the data, we chose to use a non-parametric test. Repeated measures one-way ANOVA’s were used to compare components over a treatment course. However, when the p-value of the matching was not significant in this test, we used a 2-way ANOVA.

### Software

The illustrations in Figs 1 and 6 were made by the authors using a combination of Microsoft PowerPoint, Adobe Illustrator, and Adobe Photoshop.

## Electronic supplementary material

Supplementary Information

## Data Availability

The datasets generated during and/or analysed during the current study are available from the corresponding author on reasonable request.
