# Supplementary material for: Aggregated SOD1 causes selective death of cultured human motor neurons
Source: Sci Rep. 2018 Nov 6;8:16393. doi: 10.1038/s41598-018-34759-z (PMC6219543; doi:10.1038/s41598-018-34759-z)
Supplement: Supplementary file 1 — Supplementary Information [file 41598_2018_34759_MOESM1_ESM.pdf]

Supplementary Information

Aggregated SOD1 causes selective death of cultured human motor neurons

Chen Benkler[1]*, Alison L. O'Neil[1]*, Susannah Slepian*, Fang Qian[+], Paul H. Weinreb[+], Lee L. Rubin*,[#]

[1] Contributed equally to this work
* Dept of Stem Cell and Regenerative Biology; [#] Harvard Stem Cell Institute; Harvard University, Cambridge MA

[+] Biotherapeutics and Medicinal Sciences Department, Biogen Inc., Cambridge MA
# Correspondance to lee_rubin@harvard.edu

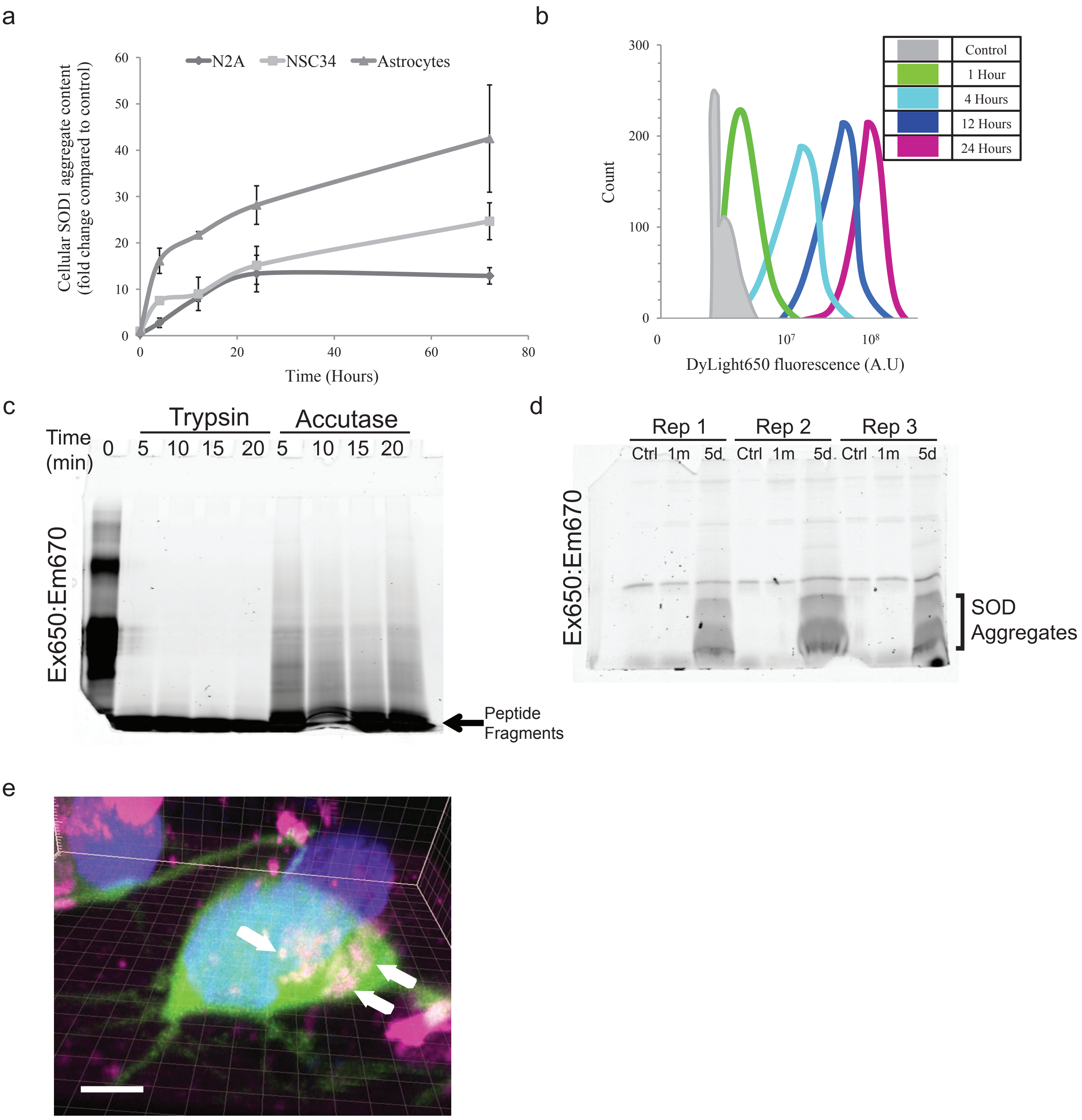

Supplementary Figure 1: Mutant SOD1 aggregates are internalized by human iPSC derived MNs.
(a) Quantified FACS analysis showing SOD1^{H46R} aggregate uptake of N2A and NSC-34 cells, as well as that of a human astrocyte cell line. The cells were treated with DyLight650 labeled SOD1^{H46R} aggregates for the indicated amounts of time, then trypsinized to remove aggregates adhering to the cell surface, uptake and accumulation were measured based on the DyLight650 labeling of the aggregates. Data are fold change in median intensity ± SD normalized to control.
(b) Representative uptake over time of Dylight650 labeled aggregates in an iPSC-derived MN culture, measured by flow cytometry. Cells were first trypsinized to remove aggregates adhering to the cell surface. A.U., arbitrary units.
(c) A representative fluorescently scanned SDS-PAGE gel comparing SOD1^{H46R}-Dylight650 aggregate digestion by 0.25% trypsin and Accutase over time. Full gel shown.
(d) A representative fluorescently scanned SDS-PAGE gel (3 technical replicates) showing that SOD1 aggregates become trypsin-resistant after incubation with cells. Full gel shown. Ctrl = untreated, 1m = 1 minute, 5d = 5 days
(e) 3D reconstruction showing SOD1 aggregates (magenta, white arrows) internalized by MNs (green). Scale bar represents 5µm.

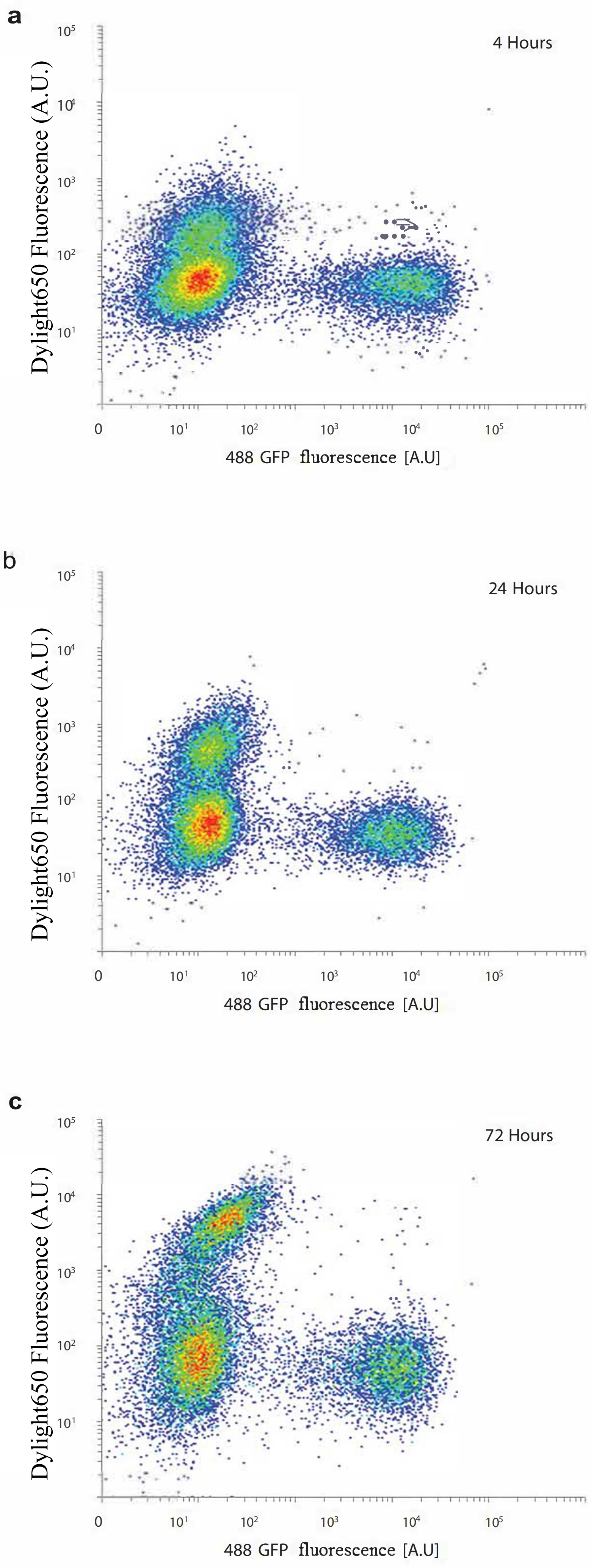

Supplementary Figure 2: Representative FACS plots of aggregate uptake.
(a) Representative FACS plots comparing 3 populations of cells based on their expression of Islet1 (x-axis, 488nm) and their extent of SOD1H46R aggregate (y-axis, 650nm) uptake over time. The two Islet1 negative populations begin to separate 4 hours after being exposed to aggregates. This pattern becomes more distinct as exposure time increases
(b) Representative FACS plot 24 hours after aggregate addition.
(c) Representative FACS plot at 72 hours after aggregate addition.

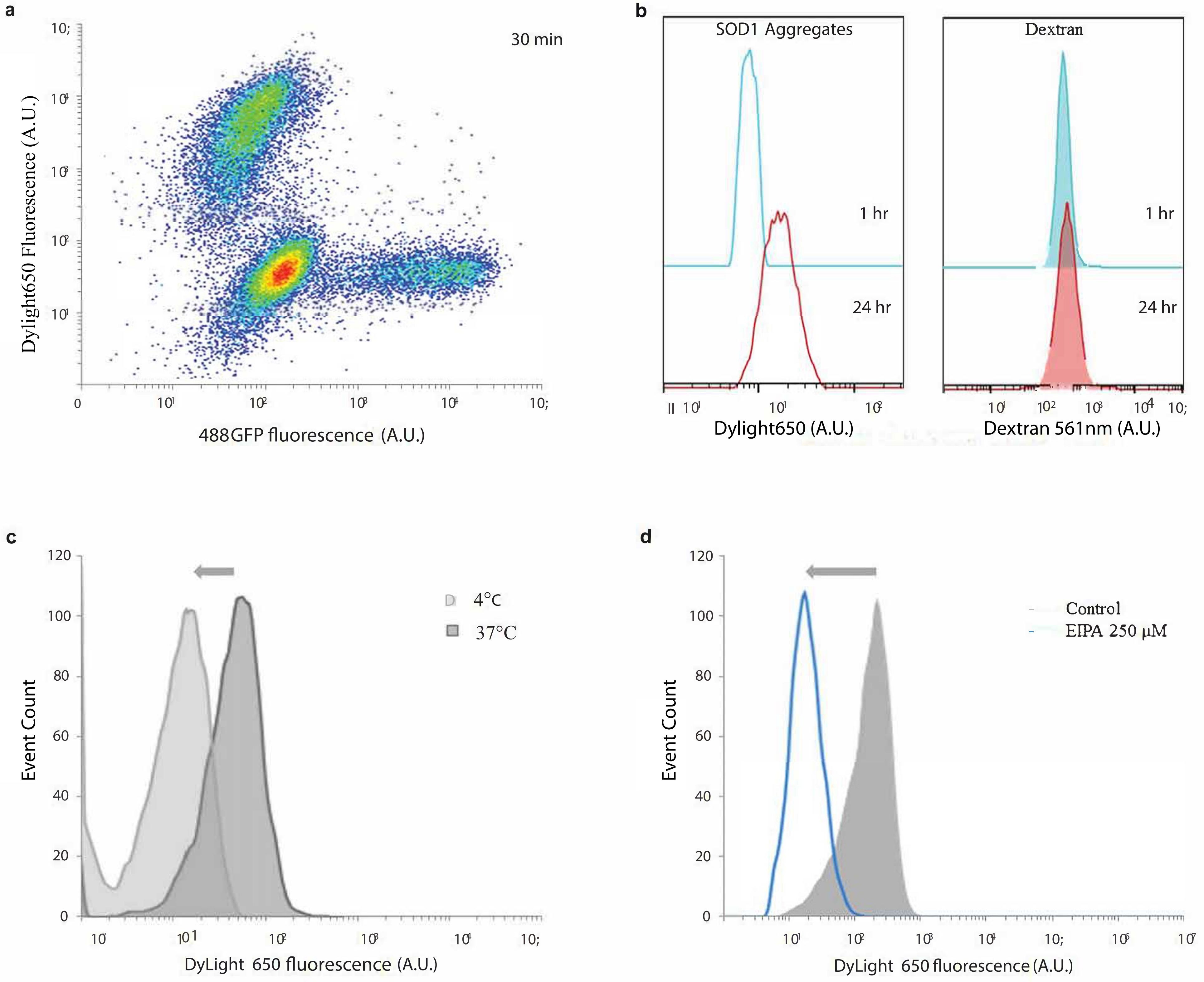

Supplementary Figure 3: $SOD1^{H46R}$ aggregate uptake and accumulation initially follows a similar rate as the fluid phase endocytosis marker dextran.

(a) A representative FACS plot illustrating the distribution of the 3 cellular populations in the iPSC-derived culture based on their expression of Islet1 (488nm) and dextran content (561nm).

(b) FACS plot of the uptake and accumulation of dextran, compared to $SOD1^{H46R}$ aggregates, which continue to accumulate over time while the dextran stays stable.

(c) FACS analysis showing inhibition of DyLight650 labeled $SOD1^{H46R}$ aggregate uptake by low temperature. The gray arrow indicates the reduction in the DyLight650 signal occurring in response to inhibition of $SOD1^{H46R}$ aggregate uptake at 4°C compared to 37°C.

(d) FACS analysis showing inhibition of DyLight650 labeled $SOD1^{H46R}$ aggregate uptake by the macropinocytosis inhibitor EIPA. The gray arrow highlights the reduction in the DyLight650 signal occurring in response to inhibition of SOD1H46R aggregate uptake.

a

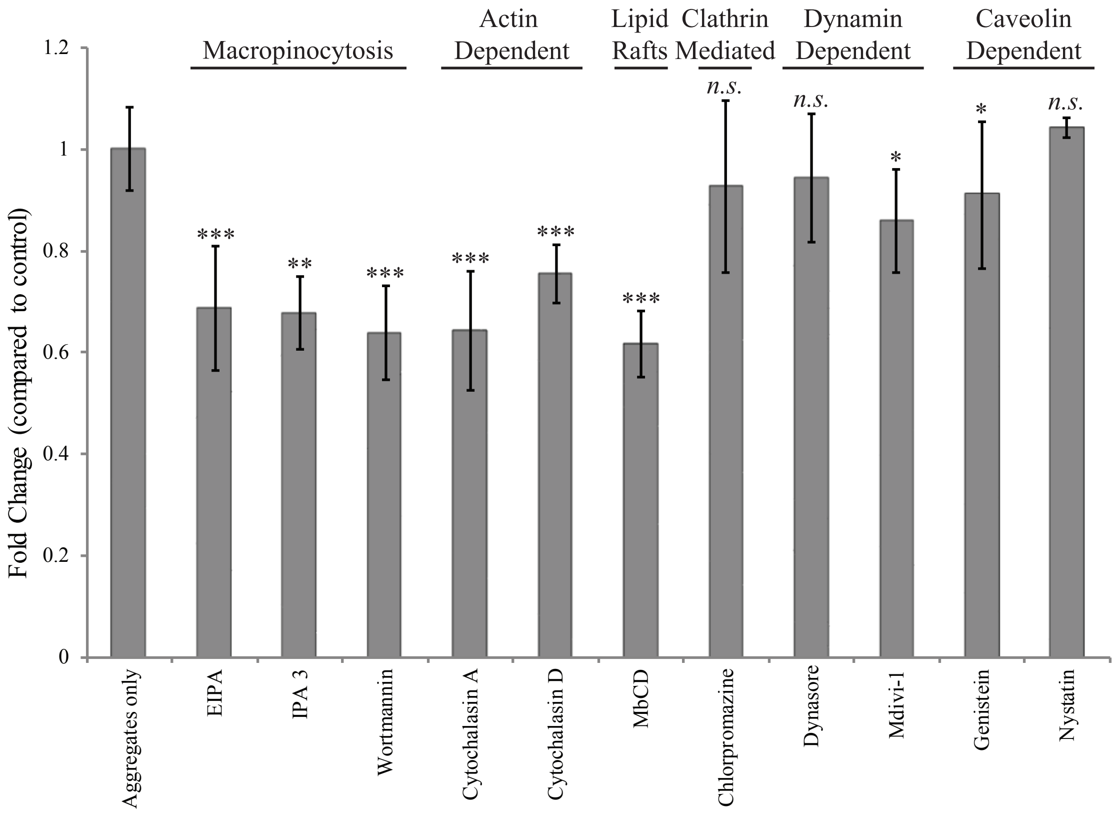

b

**Pathway**	**Treatment**	**Inhibits SOD1^{H46R} Aggregate Uptake**
Macropinocytosis	EIPA	Yes
	IPA 3	Yes
	Wortmannin	Yes
Actin Dependent	Cytochalasin A	Yes
	Cytochalasin D	Yes
Lipid Raft Dependent	MbCD	Yes
Clathrin Mediated	Chlorpromazine	No
Dynamin Dependent	Dynasore	No
	Mdivi-1	Slight
Caveolin Dependent	Genistein	Slight
	Nystatin	No

Supplementary Figure 4: SOD1 aggregate uptake is an active process mediated by endocytosis.
(a) The effect of multiple endocytosis inhibitors on SOD1^{H46R} aggregate uptake. (n=9)
(b) Table recapitulating the effects of the inhibitors used in (a). Where indicated, the human iPSC derived MNs were pretreated with the relevant inhibitors for 3 hours and then exposed to the SOD1^{H46R} aggregates for 4 hours. Data are fold change in average intensity ± SE normalized to control.
Significance was calculated using an unpaired t-test. n.s. = not significant, *$p \leq 0.05$, **$p \leq 0.01$, ***$p \leq 0.001$

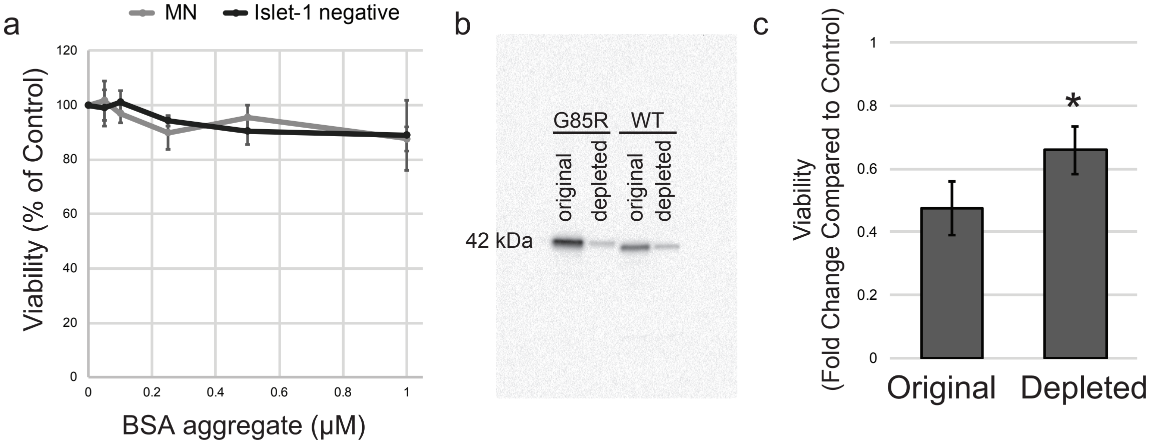

Supplementary Figure 5: SOD^{H46R} aggregates are specifically toxic.

(a) SOD1^{H46R} aggregate toxicity is specific to the SOD1 protein as aggregated BSA is not toxic to either the MNs or the Islet1 negative cells at the same concentrations. In all cases the cells were counted via Islet1-GFP+ or Hoescht and the percentages were calculated from cell numbers as compared to untreated controls.

(b) Representative Western blot showing the immuno-depletion of SOD1-YFP from the G85R and WT spinal cord lysates. Full blot shown.

(c) Quantification of the increased survival resulting from SOD1^{G85R} depletion. Data are means ± SD normalized to control. Significance was determined with an unpaired t-test, p=0.049.

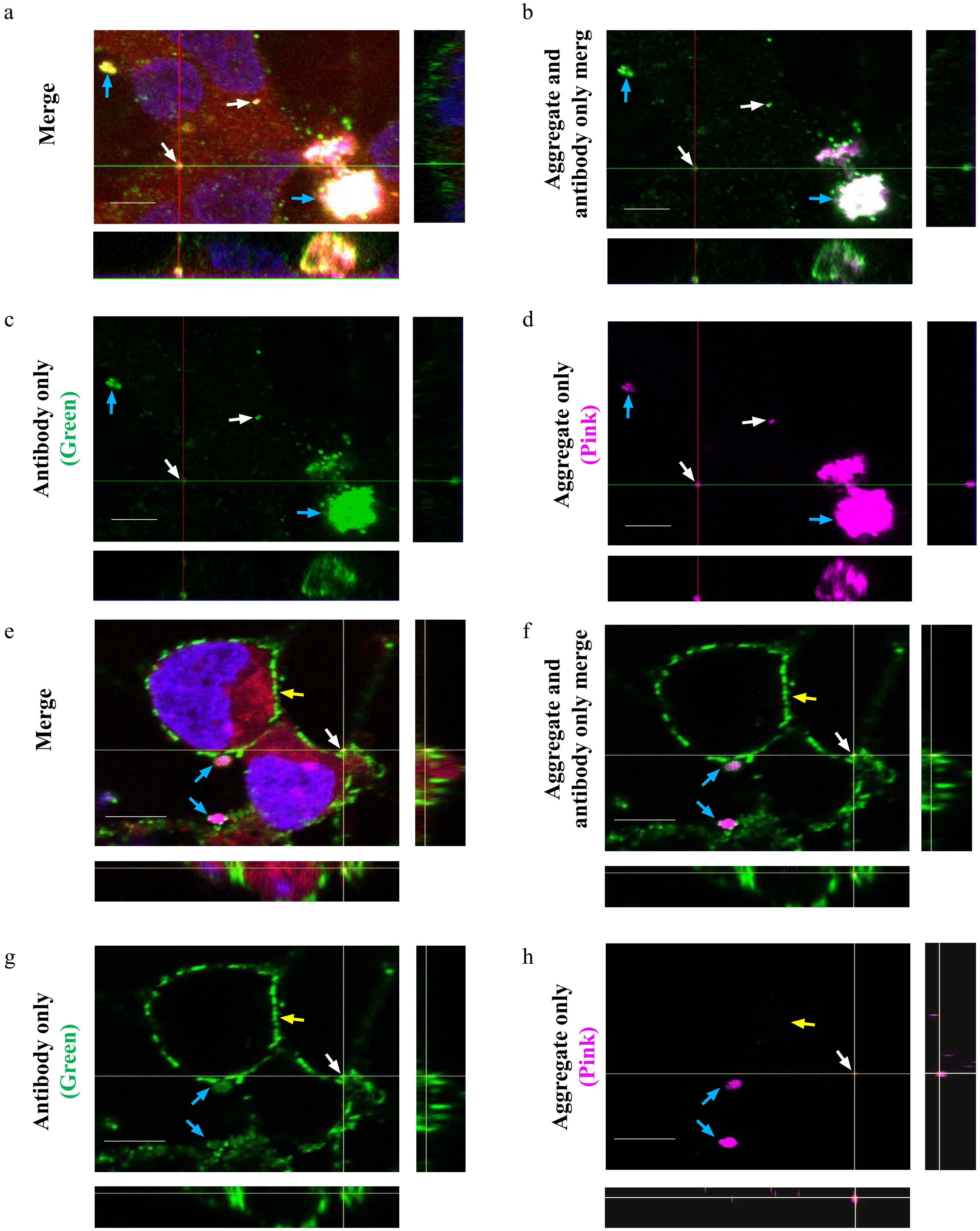

Supplementary Figure 6. αSOD1 antibodies interact with SOD1^{H46R} aggregates in several different ways.
(a-d) Confocal micrograph of motor neurons (MNs) after treatment with SOD1^{H46R} aggregates and αSOD1 antibodies at the same time.
(e-h) Confocal micrograph of MNs after exposure of the MNs to SOD1^{H46R} aggregates and αSOD1 antibodies sequentially.
The white arrows point to instances in which SOD1^{H46R} aggregates are found in association with αSOD1 antibodies inside the cell. Orthogonal views are provided to show the location of complexes in 3D space (i.e. inside the cell). The blue arrows point to instances in which an aggregate:antibody complex is found outside of the cell. In e-h, this tends to be on the cell surface. The yellow arrows highlight the different pattern of αSOD1 antibody (labeled with 488nm fluorophore in green) distribution when it is introduced sequentially versus at the same time as SOD1^{H46R} aggregates. The lipophilic dye FM 4-64FX (red) marks the cell periphery in a and e. Scale bar represents 5µm.

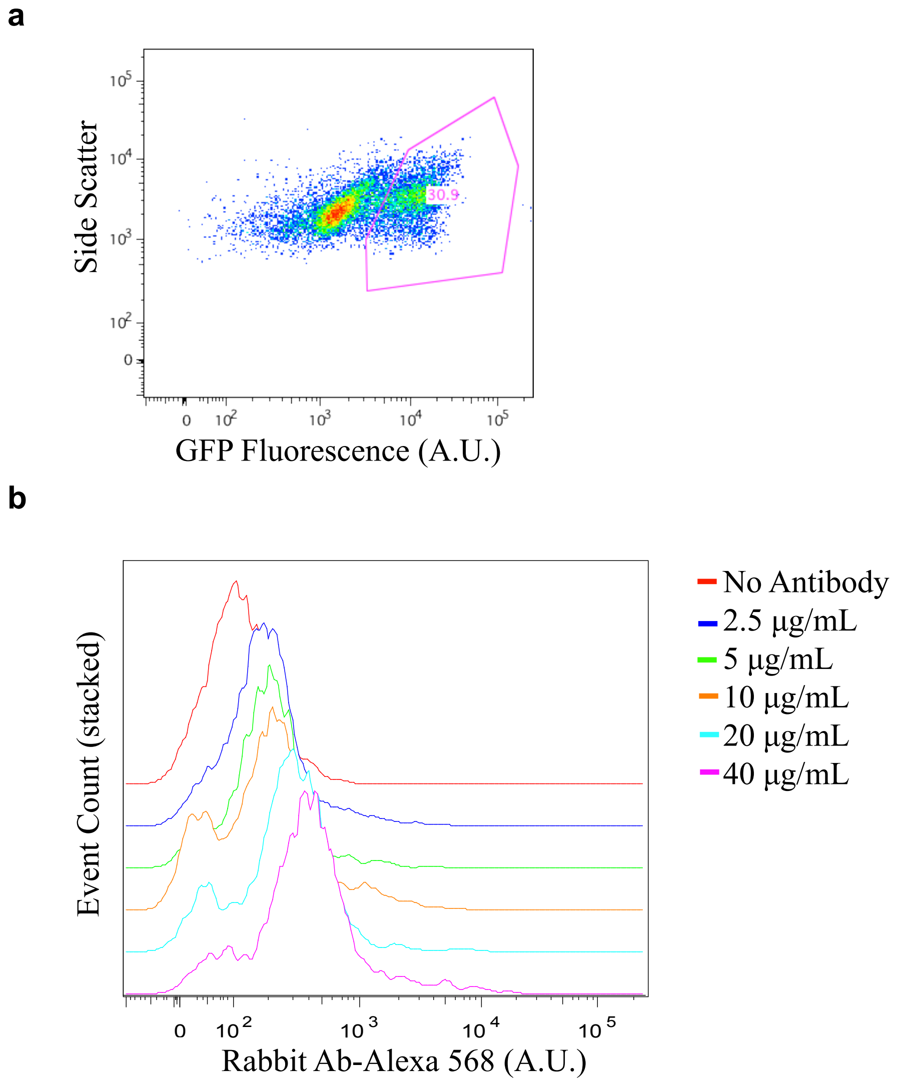

Supplementary Figure 7: Motor neurons internalize polyclonal rabbit antibodies in a dose dependent manner.

(a) Cultures were incubated with different concentrations of an Alexa 568 labeled rabbit polyclonal antibody for 3 days. Using the GFP-Islet reporter line, motor neurons were purified out of the culture (30.9% of the total population).

(b) The GFP+ motor neurons were then further analyzed for their Alex 568 fluorescence showing an increase in fluorescence corresponding to increased antibody concentration.

A.U., Arbitrary Units

Gene Name	Forward Primer	Reverse Primer
26S PSDM4	GGCCAATTGGAGGAGTTGT	GCTTTCCAACACCATCTTGC
HSP70	CAGCAGACACCAGCAGAAAA	CCTTGGATCCAGCTTGAGAG
CHOP	GATGAAAATGGGGGTACCTATG	AGGGCTAACATTCTTACCTCTTCA
BiP	CATCAAGTTCTTGCCCTTCA	TCTTCAGGAGCAAATGTCTTTGT
ATF6	CTTTTAGCCCGGGACTCTTT	TCAGCAAAGAGAGCAGAATCC
Beclin	TCACTGGGGACCTTTTTGAC	GGATCAGCCTCTCCTCCTCT
ATG5	TTCGAGATGTGTGGTTTGGA	AATGCCATTTCAGTGGTGTG
MAP2	GCTCAACATAAAGACCAGACTGC	TGGAGAAGGAGGCAGATTAGC
Islet1	AAGGACAAGAAGCGAAGCAT	TTCCTGTCATCCCCTGGATA
GAPDH	AAGGTGAAGGTCGGAGTCAAC	GGGGTCATTGATGGCAACAATA

Supplementary Table 1: List of qPCR primers used in this study.
